# Microbial flora and resistance in ophthalmology: a review

**DOI:** 10.1007/s00417-017-3608-y

**Published:** 2017-02-22

**Authors:** Andrzej Grzybowski, Piotr Brona, Stephen Jae Kim

**Affiliations:** 1 Department of Ophthalmology, Poznan City Hospital, Ul. Szwajcarska 3, 60-285 Poznan, Poland; 2 Department of Ophthalmology, University of Warmia and Mazury, Olsztyn, Poland; 3 Department of Ophthalmology, Vanderbilt University, Nashville, TN USA

**Keywords:** Antibiotic resistance in ophthalmology, Ocular microbiome, Ocular microbial flora, Ocular flora, TRUST, ARMOR

## Abstract

Antibiotic resistance in systemic infection is well-researched and well-publicized. Much less information is available on the resistance of normal ocular microbiome and that of ophthalmic infections. An understanding of the distribution of ocular microorganisms may help us in tailoring our empiric treatment, as well as in choosing effective pre-, peri- and postoperative management, to achieve the best results for patients. This study aims to summarize and review the available literature on the subject of normal ocular flora and its resistance, as well as the broader topic of antibiotic resistance in ophthalmology.

## Introduction

Ophthalmic infections vary greatly in severity. Bacterial conjunctivitis is probably the most common ophthalmic infection, often seen by primary care physicians, and is self-limiting and not sight-threatening [1]. On the other end of the severity spectrum, endophthalmitis is a rare yet extremely serious infectious complication of ocular surgery, particularly cataract surgery, ocular trauma or intravitreal injections [2].

The drug arsenal available to ophthalmologists is expected to expand continuously with the introduction of new and more effective drugs. However, this is not true in the case of antibiotics, as resistance is expected to pose an increasing threat to effective treatment of ocular infections [3].

The globe of the eye is well protected from exogenous microorganisms under normal conditions. However, penetrating trauma—be it random, surgical or associated with injection—can facilitate the migration of elements of the ocular flora into the eye, resulting in infection [4].

Although endophthalmitis is considered a very rare complication, with estimated occurrence at approximately 0.03–0.2% after cataract surgery and 0.02–0.2% after a single intravitreal injection, the high volume of cataract surgery performed worldwide results in thousands of cases of endophthalmitis each year [5, 6]. Furthermore, poor outcomes of acute endophthalmitis cause significant morbidity and vision loss globally.

The devastating effects of endophthalmitis have caused many ophthalmic surgeons to use pre- or perioperative topical antimicrobial prophylaxis. In a 2001 survey regarding antibiotic use conducted by the American Society of Cataract and Refractive Surgery, 96% of the 1300 surgeons surveyed used perioperative topical antibiotics. This remains largely unchanged today, with the more recent 2014 survey showing 90% of surgeons using perioperative antibiotics and 85% using preoperative antimicrobials [7, 8]. The choice of perioperative antibiotic depends on a multitude of factors, with major considerations including the spectrum of action, time required to eliminate bacterial flora from conjunctival surface, cost and resistance patterns [2, 3].

Identifying patients at higher risk of postoperative endophthalmitis is also important in reducing the risk of endophthalmitis [9]. Up to 82% of post-cataract endophthalmitis may be caused by ocular flora [10], underscoring the need to understand and monitor the distribution of ocular microorganisms and infections and their antibiotic resistance in order to best tailor pre-, peri- and postoperative management..

## Ocular bacterial flora

Normal ocular flora is diverse. Individual microorganisms within the ocular flora interact with each other as well as with defense mechanisms of the eye and immune system [4]. Tears function as one such antimicrobial defense—they contain the antimicrobial enzyme lysozyme, and also act together with the mechanical action of the eyelids in washing away pathogens. Under normal conditions, this results in a balance preventing the overgrowth of a particular microorganism and therefore infection [4].

Using conventional culture techniques—chocolate agar or blood agar plate, broth culture—75–82% of conjunctival cultures have been found to be positive for at least one organism [3, 11, 12].

One of the most common bacteria found on the surface of the eye is coagulase-negative staphylococci (CoNS). These are assumed to be commensal bacteria, colonizing the mucosa and lid margins [11]. CoNS are the most commonly found bacteria, detected in up to 100% of positive conjunctival cultures taken from patients preoperatively, with *Staphylococcus epidermis* the predominant species [3, 11, 13]. This has been extensively corroborated in studies from as early as 1954 [14, 15], and appears to be true around the world, with studies in Japan, Korea, the USA, Finland, Uganda and even rural populations of Sierra Leone [3, 11, 13, 16–20].

Some of the commensal organisms commonly constituting ocular flora are *Staphylococcus aureus*, *Propionibacterium*, *Corynebacterium*, *Pseudomonas aeruginosa* and *Haemophilus influenzae* [3, 11, 13]. Studies employing non-cultivable molecular techniques for determining ocular microbiome have only recently emerged [21]. In a study comparing results from conventional culture techniques and 16S RNA sequencing (a gene sequencing technique used for identifying strands of bacteria in a sample), a much wider range of microbial organisms was identified using the latter, with *Rhodococcus* sp., *Klebsiella* sp., *Propionibacterium* sp. and *Erwinia* sp. isolated [11].

Studies have attempted to identify patterns with regard to ocular flora, particularly the distribution of resistant organisms among studied populations. Research into factors affecting ocular flora may help in identifying at-risk groups and providing guidance for future prophylactic and treatment guidelines.

A high prevalence of methicillin-resistant *Staphylococcus aureus* (MRSA) has long been observed in healthcare workers [22]. However, one study investigating MRSA colonization in non-operative eyes of 399 pre-cataract surgery patients found that being a healthcare worker or family member of one did not confer additional risk of being colonized by a methicillin-resistant organism (*P* = 0.54, *P* = 0.26) [19]. This conclusion was supported by another study [23].

Researchers have reported that older patients are more likely to have MRSA or methicillin resistant CoNS, particularly those over 80 years of age, with methicillin-resistant (MR) organisms found in 29.5%, 33.3%, 34.0%, 48.3% and 50% of patients aged 50–59, 60–69, 70–79, 80–89 and 90–99 years, respectively [19]. Similar MR isolation rates have been found in patients older than 60 [13], but this correlation has not been universally reproduced, with a 2015 study of 183 preoperative eyes finding no statistically significant relation between age and colonization of MR organisms in ocular flora (*P* = 0.06) [23].

Two prospective studies in Japan both found that bacterial isolation rates were significantly lower in patients using eye drops. In one investigation of 579 eyes, the positive culture rate was 46.7% (*n* = 304) in patients not using eye drops and 30.9% (*n* = 275) in the comparative group using eye drops. Similar culture rates were observed in patients suffering from dry eye syndrome and actively using eye drops: 19.8% (*n* = 96), compared to 43.1% in those that did not (*n* = 483) [13]. A second study reported an isolation rate of 40.3% (*n* = 119, *P* < 0.001) in patients using glaucoma eye drops, versus 67.8% (*n* = 28, *P* < 0.05) in the control group not using eye drops [17]. The authors of these two studies hypothesized that these observations might be due to a washout effect following instillation of eye drops [13, 17].

Data are conflicting regarding the influence of diabetes on ocular flora. The first of the aforementioned Japanese studies found no significant difference in the bacterial detection rate in relation to diabetes status, haemoglobin A1C levels, diabetic retinopathy or glycosuria [13]. However, the study did find higher rates of methicillin resistance in patients with diabetes mellitus [13]. Similarly, a study in Turkey, designed specifically to study differences in bacteria cultured from diabetic and non-diabetic patients, found no difference in culture rates between the two groups, but did find a statistically significant (*P* = 0.018) higher rate of gram-negative organisms cultured from diabetic patients [24]. Furthermore, an investigation into the ocular flora of diabetic patients with normal and altered HbA1c levels found no difference between the two groups [25]. In contrast, a US based study from 2010, previously referenced for the data on MRSA colonization in healthcare workers, found diabetic patients to be less likely to be colonized by MR organisms (*P* = 0.02) [19]. A 2014 study based in Bangladesh showed 64% and 38% culture-positive rates for diabetic (*n* = 50) and non-diabetic (*n* = 250) patients respectively, with an additional trend of higher rates of *S. aureus* isolation in diabetic patients [26].

Another factor postulated to influence ocular flora is geographical distribution [27]. The difference in eye flora and resistance patterns in relation to geography was described as early as 1954 when results from eye cultures from two London based eye hospitals differed [14]. This is supported by a unique study of 4432 patients undergoing cataract surgery between 1994 and 1996 in Madrid, which showed significant differences in ocular surface flora that correlated to seasonal climate changes in the area [28]. The authors found that in warm, humid months—April, May and June—the overall positive bacterial rate increased. A seasonal effect was also observed with *S. pneumoniae*, with isolation rates rising in March, November and December, and with *Haemophilus sp.*, with isolation rates rising in January and April [28]. Furthermore, the authors found that the rates of rehospitalization for post-cataract extraction endophthalmitis were 3.37 times as high as those in May and June, but no statistical analysis could be performed due to a variety of confounding factors and low numbers of endophthalmitis patients overall [28].

To the best of the authors’ knowledge, no published studies have investigated geographical differences in ocular surface microbial flora between countries. Table 1 presents results from a number of studies looking into normal bacterial flora from different geographical areas.

**Table 1:** Microbial flora and percentage of positive culture results in studies from different parts of the world. Microbial levels expressed as a percentage of all positive cultures in a given study

	Influencing factor	Relation	Studies supporting the hypothesis	Studies which found no correlation
Incidence of MRSA colonization	Being a healthcare worker or immediate family of a healthcare worker	Increases likelihood of MRSA colonization		Olson et al. 2010 [19]
				Hsu et al. 2015 [23]
	Recent hospitalization	Increases likelihood of MRSA colonization		Olson et al. 2010 [19]
	Older age	Increases likelihood of MRSA colonization	Olson et al. 2010 [19]	
			Suto et al. 2012 [13]	
				Hsu et al. 2015 [23]
	Sex			Hsu et al. 2015 [23]
	Race			Hsu et al. 2015 [23]
	Diabetes	Decreases likelihood of MRSA colonization	Olson et al. 2010 [19]	
		Increases likelihood of MRSA colonization	Suto et al. 2012 [13]	
	Recent antibiotic use	Increases likelihood of MRSA colonization	Hsu et al. 2015 [23]	
Incidence of *S. aureus* colonisation	Alcoholism	Increases likelihood of *S. aureus* colonization	Gündüz et al. 2015 [29]	
	Behçet’s disease	Increases likelihood of *S. aureus* colonisation	Gündüz et al. 2008 [30]	
Culture-positive rate	Regular use of eye drops		Suto et al. 2012 [13]	
			Honda et al. 2011 [17]	
	Diabetes	Increases culture-positive rate		Suto et al. 2012 [13]
				Adam et al. 2015 [24]
			Nahar et al. 2014 [26]	
		HbA1c levels – normal versus altered		Moreno et al. 2014 [25]
	Hyperlipidemia	Lower culture-positive rate	Suto et al. 2012 [13]	
	AIDS, immunosuppression			Gritz et al. 1997 [31]
	Pregnancy and reproductive status			Balikoglu et al. 2012 [32]

Other factors that have been found to correlate with changes in the composition of ocular surface flora include alcoholism (significantly higher incidence of *S. aureus* found in subjects with chronic alcoholism compared to the healthy population) [29], Behçet’s disease (significantly higher rates of colonization with *S. aureus*, *Moraxella* sp. and *Streptococcus* sp. in Behçet’s patients) [30], and hyperlipidemia (lower bacterial detection rate, possibly due to changes in nasolacrimal duct fluid) [13].

Factors that have been investigated and found to have no effect on the composition of ocular microbial flora include AIDS and immunosuppression [31], pregnancy and reproductive status (women of reproductive age vs. postmenopausal) [32], and recent hospitalization. A summary of results from studies focusing on factors affecting ocular microbiome is shown in Table 2.

**Table 2:** Summary of research regarding factors influencing conjunctival microbial flora

Author, publication date	Olson et al. 2010 [19]	Hsu et al. 2013 [33]	Suto et al. 2012 [13]	Mshangila et al. 2013 [20]	Cavalcanti et al. 2006 [34]	Haggag et al. 2011 [35]	Capriotti et al. 2009 [16]
Study region	USA, multicenter	USA, Midwest	Japan	Uganda	Brasil	Egypt	Sierra Leone
Study population	Pre-cataract patients	Pre-cataract patients	Pre-cataract patients	Pre-cataract patients	Pre-cataract patients	Pre-cataract or pre-glaucoma surgery	Healthy individuals
Number of eyes studied	399	183	579	131	50	1000	276
Isolation rate	57%	85%	39%	46%	86%	14%	86%
CoNS	77%	74%	58%	65%	62%	76%	33%
MRCoNS	29%	34%	13%		9%	17%	No data
*S. epidermis*	64%	56%	58%	50%		76%	No data
*S. aureus*	23%	5%	4%	22%	9%	10%	23%
MRSA	2%	4%	1%		0%	4%	No data
*Corynebacterium* sp.	0%	8%	27%	0%	2%	0%	0%
Other gram-positive bacteria	5%	3%	6%	5%	16%	6%	16%
Gram-negative bacteria	6%	9%	6%	8%	11%	6%	20%
Fungi	0%	1%	0%	0%	0%	1%	30%
Comments	5% *Micrococcus* sp. found				12% *Bacillus* sp.		4.3% *Bacillus* sp., 2.5% Micrococcus

Analysis on mice suggests that ocular flora may be required for mounting a sufficient immune response to ocular infection later in life [36]. Commensal flora may thus have a more complicated role to play regarding ocular surface health and immunity. Research into the way ocular microbiome influences both innate and adaptive immunity is sparse; however, data signifying its importance continue to accumulate [37]. This is further discussed in a literature review by Kugadas et al. [37].

## Antibiotic resistance in ocular microorganisms

The development of bacterial resistance in vitro was demonstrated as early as the 1940s [33]. Since then, resistance to antimicrobial agents continues to emerge worldwide, with multidrug-resistant organisms becoming increasingly common. The mechanisms by which bacteria develop antibiotic resistance at the cellular level are mutations and genetic exchange [34]. The effects of those are further multiplied by the selective pressures in health care and community. The extended use of antimicrobials not only in hospitals, but also in long-term or day care facilities, outpatient settings, industrial livestock production and veterinary care, all promote the development and survival of resistant bacterial strains [34].

Infections caused by antimicrobial-resistant strains may not only be more difficult to treat, but can also cause increased morbidity. An experimental study on rabbits found endophthalmitis caused by resistant *S. epidermidis* caused more inflammation and ocular tissue destruction than non-resistant strains [35].

Emergence and progression of resistance on a regional, national, and worldwide scale has been widely studied and is almost universally accepted. As listed above there is a multitude of factors influencing development of resistance. In view of the multifactorial nature of antibiotic resistance, a very important question, in terms of shaping future practice, is whether we as ophthalmologists can influence the resistance patterns of our patients with our daily practice. At least two studies have demonstrated that use of prophylactic antibiotics in the setting of intravitreal injections causes a statistically significant rise in ocular colonization with resistant strains [38–40].

In a study by Milder et al., 80 eyes from 40 patients who had previously received at least three injections for exudative AMD in one eye only were selected, the other eyes serving as controls. Patients had received seven injections on average (range 3–13) in the study eye, and were given a single drop of fluoroquinolone and either polymyxin B/trimethoprim eye drops (*n* = 29) or fluoroquinolone eye drops (*n* = 11) for 4 days afterwards [40]. The rate of resistance to fluoroquinolones was almost double the resistance in the controls (63.6% vs 32.1%, *p* = 0.04). Furthermore, among eyes treated with a 4-day post-injection course of fluoroquinolone, resistance was 87.5% (*n* = 8), compared with 25% in the matched untreated eyes (*p* = 0.04) [40]. No difference in trimethoprim resistance was found.

In a prospective randomize, longitudinal study by Kim and Toma, 48 eyes from 24 patients undergoing unilateral intravitreal injections were selected, with contralateral eyes not receiving injections serving as matched controls [41]. These patients were then randomized to the use of either ofloxacin, gatifloxacin, moxifloxacin or azithromycin (8 patients in each group), and using only their assigned antibiotic after each injection. Injections were administered 4 weeks apart, and patients were instructed to use their antibiotic for 4 days after the injection, 4 times a day for fluoroquinolones and 2 times a day for azithromycin. Baseline resistance of CoNS to erythromycin and azithromycin was 57% and 65%, respectively, and resistance to fluoroquinolones ranged from 34 to 39% for moxifloxacin and gatifloxacin, and from 57% to 52% for ofloxacin and levofloxacin [41]. A total of 70 CoNS isolates were identified from control eyes; those did not demonstrate a significant increase in rates of resistance to fluoroquinolones or macrolides over the study period. In eyes treated with fluoroquinolones, 48 CoNS cultures were grown (visits 1–4), and showed ofloxacin and levofloxacin resistance of roughly 85% (*p* = 0.003), and resistance to gatifloxacin and moxifloxacin approaching 67% (*p* = 0.009) and 77% (*p* < 0.001), respectively [41]. A similar trend was found in eyes treated with azithromycin, with resistance to macrolides of 94% (*p* = 0.009, compared to fluoroquinolone-treated eyes), along with decreased levels of resistance to fluoroquinolones [41].

Finally, Hsu et al. studied changes in conjunctival flora and resistance patterns in patients undergoing intravitreal injections without post-injection antibiotics, relying on povidone-iodine antisepsis only. The study concluded that no significant changes to ocular flora or resistance patterns occurred in studied subjects [42]. This further supports that the causative factor in two previously described studies is most likely antibiotic use. The three above aforementioned studies interpreted collectively suggest that antibiotic use may have a measurable and immediate influence in the emergence of resistant bacterial strains in our patients.

In response to a perceived threat from increasing antibiotic resistance worldwide, the World Health Organization, the United States Food and Drug Administration, and other large organizations started surveillance programs amalgamating data from the USA and worldwide [43–45].

Two such initiatives are of particular interest to ophthalmology—Ocular Tracking Resistance in the U.S. Today (TRUST) and Antibiotic Resistance Monitoring in Ocular Microorganisms (ARMOR) [43, 44, 46].

TRUST is a nationwide US-based multicenter surveillance program established in 1996, in which isolates are sent from over 200 clinical laboratories to an independent central laboratory for in vitro susceptibility testing. An ocular-specific substudy was initiated in 2005 (Ocular TRUST1) looking to gather prospective data each year as well as to retrospectively analyze ophthalmic samples from previous years [44, 46]. The TRUST study looks specifically at three microorganisms—*Staphylococcus aureus*, *Streptococcus pneumoniae*, *Haemophilus influenzae. S. aureus* being further divided as methicillin-susceptible (MSSA) or methicillin-resistant (MRSA) [44, 46].

The ARMOR study is a similar surveillance program set up specifically to monitor ocular pathogens across the United States. Initial results from the ARMOR study based on isolates collected from 34 institutions over the course of 2009 were published in 2011 (ARMOR 2009), and subsequent data from 2009 through 2013 (ARMOR 2013) were published this year. The ARMOR study extends data collected for TRUST studies with analysis of *Pseudomonas aeruginosa* and CoNS. The ARMOR 2013 study analyzed a total of 3237 isolates and is the largest study of its kind to date.

Table 3 provides a summary of the resistance levels found in the TRUST and ARMOR studies.

**Table 3:** Percentage of non-susceptible bacteria (intermediate and high-level resistance) in TRUST and ARMOR studies

Bacteria		Penicillin	Azithromycin	Gatifloxacin	Moxifloxacin	Levofloxacin	Ofloxacin	Tobramycin	Ciprofloxacin
*Streptococcus pneumoniae*	TRUST retrospective	34.1%	33.4%	03%	0.1%	0.1%		95.1%	9.7%
	TRUST prospective	18.3%	22.4%	0%	0%	0%		98%	10.2%
	ARMOR	31.8%	34.8%	0.4%	0.3%	0%	0.4%		
MS *Staphylococcus aureus*	TRUST prospective	90.2%	45.7%	18.9%,	18.9%	18.9%		7.3%	20.1%
	ARMOR		42.8%	13.5%	12%	13.7%	14.1%	4.1%	14.2%
MR *Staphylococcus aureus*	TRUST prospective	100%	93.9%	84.8%	84.8%	84.9%		63.6%	84.8%
	ARMOR		93.3%	75.1%	74%	75.9%	76.4%	44.3%	77.3%
MS coagulase-negative staphylococci	ARMOR		44.7%	13.9%	13.6%	13.9%	14.2%	6.4%	15%
MR coagulase-negative staphylococci	ARMOR		78.3%	55.7%	51.2%	56.8%	56.9%	23.1%	58.5%
*H. influenzae*	TRUST retrospective	100%	0.3%	0.3%	0.3%	0.3%		0%	0.3%
P. aeruginosa	ARMOR					6.9%	10.1%	3.1%	7.7%

## Difficulties in assessing resistance in ophthalmology

Studies regarding antibiotic resistance in ophthalmological practice have often been confounded by small case numbers [44]. This is especially apparent in the specific case of microbiological isolates from cases of endophthalmitis, as the low incidence rate implies that single-center studies are unlikely to gather sufficient data for results to be statistically significant [43, 44]. Although the Ocular TRUST and ARMOR 2009 studies included a large number of prospectively gathered isolates, it was not until the ARMOR 2013 results were published that trends in resistance could be statistically analyzed.

Another limitation of published studies lies in the way bacterial susceptibility is detected. Determination of bacterial resistance is based on minimum inhibitory concentrations (MICs), the lowest concentration of an antimicrobial that will inhibit growth of a given microorganism. The MICs used in most studies are based on systemic drug administration, and subsequently on the average concentrations of the drug in tissues [3, 13, 43, 44, 46]. This is not necessarily representative of antimicrobial therapy used in the treatment of patients. For example, topical application of antimicrobials, as commonly used in ophthalmological practice is likely to provide higher drug exposure over time than systemic use [44]. As noted in Ocular TRUST1, one study looking into pharmacokinetics of 0.5% levofloxacin ophthalmic solution found that the area under the curve (AUC, a measure of drug exposure time) over 6 h was more than twice that with oral or intravenous dose of 750 mg levofloxacin over 24 h [44, 47]. Similar findings have been reported for azithromycin with application of 0.5%, 1% or 1.5% azithromycin topical solution, resulting in tear film AUC0-24 (a measure of drug exposure over 24 h) of between 108 and 362, two orders of magnitude higher than standard oral 3- or 5-day regimens or single-dose extended release (AUC0-24 of 2.58, 2.60, and 8.62), respectively [48, 49]. The effect of higher antibiotic concentration with topical application may mean that thresholds set for resistance in large databases such as the Clinical Laboratory and Standards Institute (CLSI) or automatic microbial equipment underestimate antimicrobial sensitivity in ocular pathogens.

One more difficulty with correlating laboratory data with clinical effectiveness arises from difficulties in obtaining data regarding penetration of topical antibiotics into deeper structures of the eye and subsequently their concentrations over time. Antibiotic penetration may be another factor influencing clinical success rates, but there are limited data on this subject. One study found that topical moxifloxacin and gatifloxacin penetrated the anterior chamber to a greater degree than ciprofloxacin, achieving much higher concentrations [50].

Another issue that makes accurate assessment of resistance difficult is the lack of a standardized framework for studying ocular pathogens and the different interpretive criteria for susceptibility. Although some, like the aforementioned trials, used CLSI-defined breakpoints [3, 13, 43, 44, 46], others used automatic microbial systems [51], and some studies reported the method of testing or the laboratory that performed it but not the framework adhered to or thresholds that were chosen [19, 52].

A potential weakness present in both Ocular TRUST1 and ARMOR is selection bias. The methodology for both studies is based on analyzing samples of already cultured organisms [44, 46]. However, culturing in ophthalmology is relatively infrequent, and although treatment guidelines for many types of ophthalmic infections state that culture should be taken prior to commencement of antibiotic therapy, clinicians will sometimes start empiric therapy and take cultures only if the therapy fails [2]. Additionally, cultures are more likely to be taken if the severity of infection is greater. Overall, this might skew the results towards representing more severe and potentially more resistant infections. Finally, both studies focused solely on microbial isolates of pre-defined taxonomy, omitting a spectrum of antibiotic resistance in other pathogenic bacteria.

## Resistance levels in TRUST and ARMOR studies

### Staphylococcus pneumoniae

Retrospective analysis of 760 *S. pneumoniae* archived samples gathered between 1999 and 2006 in the TRUST study has shown 34.1% of *S. pneumoniae* to be penicillin-resistant, with nearly three-quarters of those being cross-resistant to azithromycin and trimethoprim [44]. The data gathered prospectively by the Ocular TRUST1 study between 2005 and 2006 revealed 9 of 49 (18.3%) *S. pneumoniae* isolates to be resistant to penicillin, with all of those also resistant to trimethoprim, azithromycin and tobramycin [44]. Only a single isolate of the 760 was resistant to third- and fourth-generation fluoroquinolones (levofloxacin, moxifloxacin, gatifloxacin). Ciprofloxacin resistance was 9.7%. There were no statistically significant changes to *S. pneumoniae* fluoroquinolone susceptibility over the 8 years of the study [44].

ARMOR 2013 further corroborates these data, with penicillin resistance at 31.8%, azithromycin resistance at 38.4%, and only single isolates resistant against any generation of fluoroquinolones [46].

### Pseudomonas aeruginosa

Resistance rates among *P. aeruginosa* samples were low against all antibiotics tested in ARMOR 2013. With the exception of polymyxin B, susceptibility was above 90%.

### Haemophilus influenzae

Of the 356 *H. influenzae* isolates gathered in the retrospective arm of Ocular TRUST1 (1999–2006), 37.4% were β-lactamase-positive. However, all *H. influenzae* samples were susceptible to penicillin and all other antibiotics tested, with the exception of 14.3% resistance to trimethoprim [44]. In the prospective portion of Ocular TRUST1, β-lactamase-producing isolates accounted for 44% of samples (14 of 32); this had no impact on antibiotic resistance [44]. The ARMOR study has further supported those findings, showing all but two isolates to be susceptible to all antibiotics tested in 2009 (*n* = 73) and in the following 4 years (*n* = 284), the two exceptions being a single isolate resistant to chloramphenicol and another to chloramphenicol. Neither β-lactamase production nor trimethoprim susceptibility was tested [43, 46].

### Coagulase-negative staphylococci and Staphylococcus aureus

The prospective arm of Ocular TRUST1 shows 83.2% of *S. aureus* isolates to be methicillin-susceptible. Most MSSA isolates were found to be susceptible to fluoroquinolones, with less than 20% resistance for any of the fluoroquinolones tested [44]. This is contrasted by the 75–85% resistance against fluoroquinolones tested in MRSA isolates. The only agent consistently active against both MSSA and MRSA was trimethoprim, showing only 6.4% resistance in MRSA and 2.4% in MSSA [44]. The data from the ARMOR 2009 study showed 39% of *S. aureus* isolates to be methicillin-resistant, compared to the 16.8% from Ocular TRUST1 isolates gathered in 2006 [43, 44]. Similar to the results found in Ocular TRUST1, MRSA isolates were found to be multi-resistant, with 79.5% resistance to ciprofloxacin, 65.4% resistance to moxifloxacin, and 52.6% resistance to tobramycin [43]. Although MRSA resistance to fluoroquinolones did not increase between the two studies, the doubling of MRSA incidence among *S. aureus* isolates may mean that ocular infections caused by *S. aureus* are twice as likely to be caused by a methicillin-resistant—and possibly multi-resistant—pathogen. In the ARMOR 2009 study, 11.5% of *S. aureus* isolates were not susceptible to five different drug classes [43]. In the ARMOR 2013 study, 86.8% and 77.3% of MRSA and methicillin-resistant CoNS, respectively, were found to be resistant to three or more drug classes.

Among CoNS isolated in ARMOR 2013, the vast majority consisted of *S. epidermis* (75.9%), perhaps reflecting how commonly this species forms part of the commensal flora [46]. The resistance rates from the ARMOR 2013 study showed that the CoNS resistance profile follows trends similar to *S. aureus*, with resistance rates of 49.7% to methicillin (42.2% for *S. aureus*), 34.4% to ciprofloxacin (39.8% for *S. aureus*), and 61.3% to azithromycin (63.6% for *S. aureus*). Perhaps surprisingly, no statistically significant rise in CoNS or *S. aureus* resistance was detected during the 5-year surveillance period of the ARMOR 2013 study [46].

The latest preliminary reports from the ARMOR study for 2014 and 2015 show that high levels of resistance in ophthalmology, including multi-drug resistance, continue to be a reality and a challenge today [53].

### Preventing the spread of resistance in ophthalmology

#### Subtherapeutic dosage

A key facet of antimicrobial prescribing is ensuring that we use antimicrobials in the correct dosage and for the proper length of treatment. Using too low a dose—so called subtherapeutic dosage—can accelerate the development of drug resistance: it exposes the microbes to the drug without killing them, allowing them to develop resistance, multiply and spread. Similarly, resistance can be promoted with antimicrobial treatment duration that is too short. Incorrect dosages for antimicrobials are surprisingly common, especially for children, as many drugs are not available in pediatric dosages. A study in 2015 showed that nearly half the children in the sample were treated with suboptimal dosages of commonly used antifungal agents [54]. A possible driver of this routine non-optimal dosing is the lack of recent studies on the pharmacokinetics (PK) and pharmacodynamics (PD) of antibiotics. Additional studies would enable a better understanding of how the drug is broken down, absorbed, and excreted. This is crucial for determining optimal dosing regimens. Many PK and PD studies on antibiotics were conducted in the 1950s and 1960s, when these antibiotics were first discovered. Now, with improved techniques and protocols for PK/PD studies, these antibiotics must be re-evaluated to ensure that they are being used in the most efficient way possible. Many of the antibiotics used in ophthalmology in particular are topical or intracameral, and thus most of the studies regarding systemic administration PK/PD parameters may not be applicable—and may even be misleading.

#### Using the right antibiotic

In an ideal world, prescribing of an antibiotic would occur only after identification of the pathogen, along with resistance testing, in order to ensure the choice of the best-suited antimicrobial. In real world practice this is not always practical or even possible, so antibiotics are often prescribed empirically. One of the key issues with conventional culture and disk diffusion or broth dilution resistance testing is the relatively long time before the pathogen is identified (24–48 h), and another 24 h to complete susceptibility testing [55]. By the time a clinician receives the full culture report, the response to the antibiotic, started 48–72 h earlier, can often be judged empirically by examining the patient. To address this problem, a number of rapid diagnostic tests are being developed and employed [55]. These include many polymerase chain reaction (PCR) and peptide nucleic acid fluorescence in situ hybridization (PNA-FISH) tests from various manufacturers [55]. Both of these tests work by identifying known resistance or species-specific coding sequences. The PCR method amplifies the sought-after sequence, making it detectable by other methods such as electrophoresis, while PNA-FISH uses fluorescence for detection. Results from these tests are available within 45 min to 6 h [55]. Another emerging method for rapid susceptibility testing is bacterial cytological profiling (BCP). BCP relies on identifying key changes in pathogens exposed to a particular antibiotic by assessing individual cytological parameters of hundreds of cells using automated systems [56]. The cultured pathogens are treated with an antimicrobial, and the response in terms of cell and nucleus size, shape and volume is assessed under fluorescence-based microscopy for specific changes indicating susceptibility or resistance [56]. It is important to note that neither of these methods is being developed with ophthalmology in mind, aiming instead at aiding in the diagnosis of systemic infections. Thus they would need to be adapted for ophthalmic use. In the future, these innovations may help ophthalmologists in choosing the right antimicrobial and reserving antibiotics of last resort, while remaining confident in the therapeutic effect of the antibiotic prescribed.

#### Using antibiotics only when proven to provide a tangible benefit

An indispensable message in every antibiotic stewardship program is the importance of using antibiotics only when necessary [57]. The case of antimicrobial prophylaxis in the setting of IVI is a prime example where research has shown that alternative infection control methods are at least as effective [6]. An additional benefit in not using antibiotic prophylaxis in IVI is an estimated annual savings of $300 million in the USA, compared to using antibiotic prophylaxis for every IVI [58]. A second area where a large-scale change in practice may be possible is the use of topical antibiotics prior to cataract surgery. A recent review of the literature concluded that, despite the widespread practice, the evidence supporting preoperative topical antibiotics is not compelling [59]. Finally, antibiotics are often misused in the treatment of viral and allergic conjunctivitis [58]. Studies show that up to 80% of conjunctivitis cases are of viral origin, which do not require treatment with antibiotics and are usually self-limiting [60]. Moreover, topical antibiotics are contraindicated, as they do not protect against secondary infections, and may blur the clinical presentation by causing toxicity or allergic reactions [60]. This is a problem that has been highlighted in the past, with the American Academy of Ophthalmology designating antibiotic use in the setting of IVI and viral conjunctivitis as two of the top five unnecessary interventions in ophthalmology [58].

#### Alternatives to antibiotics

Previously there were hopes that the newer-generation fluoroquinolones might help stop the spread of resistance [2]. Today, with reports of resistance levels to those antibiotics as high as 70%, this hope appears unfulfilled [38]. There are, however, important alternatives to antibiotics that are applicable in many situations. A recent review of upcoming antibiotic substitutes or adjuncts highlighted 10 key emerging approaches, including immune stimulation, probiotics, lysins, bacteriophages and antimicrobials peptides [61]. Although this review was focused on systemic therapy, antimicrobial peptides were identified as having considerable potential for topical therapy [61].

One such alternative already employed in ophthalmology is the use of effective antiseptics, as illustrated in the case of IVI. In recent years, large-scale research has demonstrated the safety of using povidone-iodine 5% solution in preventing IVI-associated endophthalmitis [62]. A recent survey of American retina specialists has shown that 89% do not use any antibiotics in IVI, and another 5% use them only in select patients, reflecting major antibiotic-sparing initiatives in ophthalmology are still very much possible in this day and age [63]. The hope is that, with current and future reports coming from the USA of good outcomes without the use of antibiotics, the rest of the world will gradually adopt an IVI practice based on good aseptic techniques, thereby minimizing the use of topical antibiotics and reducing the rise of antimicrobial resistance.

Antiseptics have become a mainstay in postoperative endophthalmitis prevention. Povidone-iodine (PVI) and chlorhexidine are the two major antiseptics of choice in ophthalmology. PVI solutions act by releasing free iodine, which readily penetrates the microbial membrane, causing intracytoplasmic oxidation of proteins and cell death as a result [64]. Chlorhexidine, on the other hand, is a much larger molecule and cannot penetrate the microbial cell wall—depending on the concentration, it exerts a bacteriostatic effect, with destruction of cell membranes. At higher concentrations it shows bactericidal activity by causing leakage, coagulation, and precipitation of cellular contents [64]. Although both antiseptics offer good activity against a large spectrum of gram-positive bacteria, PVI has a larger spectrum against other microorganisms. The spectra of activity of the two antiseptics are compared in Table 4. Although both PVI and chlorhexidine can cause hypersensitivity reactions, there have been no reports of anaphylaxis following topical application of PVI [65]. The allergy profile of PVI is considered excellent; adverse skin reactions to topical PVI are very rare and are overwhelmingly caused by skin irritation rather than allergic immunological processes [66]. Immunological reactions to chlorhexidine are comparatively common, and relatively frequent allergic contact dermatitis as well as urticarial and anaphylactic reactions have been described [66]. Unfortunately, the phenomenon of resistance is not limited to antibiotics. Chlorhexidine resistance has been described, particularly among MRSA and other staphylococcal infections, with a number of responsible genes identified [67]. To the best of the authors’ knowledge, there are no reports to date of PVI resistance. A recent work showed that PVI 1.25% was an effective alternative to antibiotics in treating bacterial keratitis, suggesting that antiseptic use in ophthalmology may expand beyond prevention [68].

**Table 4:** Spectra of antimicrobial activity for povidone-iodine and chlorhexidine. Adapted from Lachapelle et al. [64]

		Chlorhexidine	Povidone-iodine 10%
Gram-positive bacteria	Activity	High	High
	Spectrum	Large	Large
Gram-negative bacteria	Activity	High	High
	Spectrum	Incomplete	Large
Fungi	Activity	Medium	High
	Spectrum	Incomplete	Large
Viruses	Activity	Low	Medium
	Spectrum	Incomplete	Large
Actinobacteria	Activity	No activity	Medium
Spores	Activity	No activity	Medium

## Conclusions

The eye surface is home to a diverse set of organisms. Based on current information, we have a good understanding of what microbes normally constitute the ocular biome. Nevertheless, much remains to be learned regarding factors influencing the composition, characteristics, resistance and pathogenicity of ocular flora, in order to effectively combat resistance in ophthalmology. The WHO, in its *Global Action Plan on Antimicrobial Resistance*, outlines a few strategic objectives which relate to the current situation in ophthalmology [69]. WHO notes that, in order to fight resistance, important gaps in knowledge, particularly the “…incidence, prevalence, range across pathogens and geographical patterns related to antimicrobial resistance is needed to be made accessible in a timely manner….” More work remains to be done on the geographical distribution of both normal flora and resistance in normal flora and infection.

Effective antimicrobials are needed in many preventive and curative efforts in ophthalmology. However, distinguishing which antibiotics should be used in what situations, and where alternatives to antibiotics are more appropriate, is critical. Intravitreal injection (IVI) is a prime example where unnecessary and/or improper use of antibiotics may have serious consequences.

Where antibiotics are required, they should be used in accordance with established microbiological guidelines and specifications in order to obtain high concentrations in target tissue and maintain sufficient duration of effects to reduce resistance. Repeated short-term exposure to topical antibiotics, as is seen in the setting of IVI for chronic retinal disease, can quickly promote an antibiotic-resistant ocular biome. This is illustrated by reported rates of resistance to moxifloxacin and gatifloxacin of as high as 70% in patients undergoing IVI with antibiotic prophylaxis after just 1 year of serial IVI [38].

Equally important, we as clinicians should be aware that labeling a microorganism as resistant is based on both systemic administration of antibiotics and systemic infection. Concentrations achieved through topical use are often much higher and may still be effective.

Microbial resistance is an important subject, with much innovation and research in prevention, detection and treatment of resistant bacteria. Unfortunately, for ophthalmologists, most of this research is focused on systemic infection, and it will take some time before diagnostic and preventive methods are viable in ophthalmology. Furthermore, many may never be validated for ophthalmic use. The situation is different with antiseptics—PVI and chlorhexidine are already used in everyday practice, and their use may expand to replace antibiotics in procedures other than IVI. Most importantly, both antiseptics have key advantages of non-selective mechanisms of action (preventing the development of resistance) and low cost.
